# Beyond Pathogen Filtration: Possibility of Smart Masks as Wearable Devices for Personal and Group Health and Safety Management

**DOI:** 10.2196/38614

**Published:** 2022-06-21

**Authors:** Peter Lee, Heepyung Kim, Yongshin Kim, Woohyeok Choi, M Sami Zitouni, Ahsan Khandoker, Herbert F Jelinek, Leontios Hadjileontiadis, Uichin Lee, Yong Jeong

**Affiliations:** 1 KAIST Institute for Health Science and Technology Korea Advanced Institute of Science and Technology Daejeon Republic of Korea; 2 Graduate School of Data Science Korea Advanced Institute of Science and Technology Daejeon Republic of Korea; 3 Information & Electronics Research Institute Korea Advanced Institute of Science and Technology Daejeon Republic of Korea; 4 Department of Biomedical Engineering Khalifa University of Science and Technology Abu Dhabi United Arab Emirates; 5 Healthcare Engineering Innovation Center Khalifa University of Science and Technology Abu Dhabi United Arab Emirates; 6 Department of Electrical and Computer Engineering Aristotle University of Thessaloniki Thessaloniki Greece; 7 School of Computing Korea Advanced Institute of Science and Technology Daejeon Republic of Korea; 8 Department of Bio and Brain Engineering Korea Advanced Institute of Science and Technology Daejeon Republic of Korea

**Keywords:** smart mask, pathogen filtration, COVID-19, protective equipment, digital health, wearable, smart device, wearable device, sensor, health monitoring

## Abstract

Face masks are an important way to combat the COVID-19 pandemic. However, the prolonged pandemic has revealed confounding problems with the current face masks, including not only the spread of the disease but also concurrent psychological, social, and economic complications. As face masks have been worn for a long time, people have been interested in expanding the purpose of masks from protection to comfort and health, leading to the release of various “smart” mask products around the world. To envision how the smart masks will be extended, this paper reviewed 25 smart masks (12 from commercial products and 13 from academic prototypes) that emerged after the pandemic. While most smart masks presented in the market focus on resolving problems with user breathing discomfort, which arise from prolonged use, academic prototypes were designed for not only sensing COVID-19 but also general health monitoring aspects. Further, we investigated several specific sensors that can be incorporated into the mask for expanding biophysical features. On a larger scale, we discussed the architecture and possible applications with the help of connected smart masks. Namely, beyond a personal sensing application, a group or community sensing application may share an aggregate version of information with the broader population. In addition, this kind of collaborative sensing will also address the challenges of individual sensing, such as reliability and coverage. Lastly, we identified possible service application fields and further considerations for actual use. Along with daily-life health monitoring, smart masks may function as a general respiratory health tool for sports training, in an emergency room or ambulatory setting, as protection for industry workers and firefighters, and for soldier safety and survivability. For further considerations, we investigated design aspects in terms of sensor reliability and reproducibility, ergonomic design for user acceptance, and privacy-aware data-handling. Overall, we aim to explore new possibilities by examining the latest research, sensor technologies, and application platform perspectives for smart masks as one of the promising wearable devices. By integrating biomarkers of respiration symptoms, a smart mask can be a truly cutting-edge device that expands further knowledge on health monitoring to reach the next level of wearables.

## Introduction

After the World Health Organization declared COVID-19 a “pandemic” (a global epidemic) attributed to SARS-CoV-2 infection [1], masks have been used by the general population all over the world for precautionary health reasons [2,3]. As a result, people wear masks at all times and in all places; however, the pandemic has revealed the limitations of current mask deployments regarding not only the spread of the disease but also concurrent psychological, social, and economic complications.

To improve these limitations, smart face masks designed with electronic sensors have been recently proposed. The continuous use of masks has led to the designs of various face mask products, which have become available on the market. The term “smart” has been used to signify possible additional functionalities of the “smart (face) masks” around the world, leading to an expansion of the mask’s usage, including masks for protection, health, and environmental sensing [4-6]. 

While the COVID-19 pandemic is seemingly under control owing to vaccination, there is a need for innovative, Internet of Things (IoT)–based smart-mask solutions to help people transition to a postpandemic world, where the emergence of infectious SARS-CoV-2 variants is prevalent along with the heightened possibility of further, yet unknown, virus pandemics, and to combat airborne diseases [7,8]. In combination with data-driven applications, IoT and smart connected technologies can play a critical role in individual protection and extend to group sensing for the prevention, mitigation, and continuous remote monitoring of patients. Such a benefit of group sensing is shown with a contact-tracing app, where it could instruct a person in close contact with patients with COVID-19 to quickly self-isolate to reduce disease transmission [9].

Here we present a viewpoint for smart masks in the form of emerging IoT-based solutions by examining the current status of smart masks, potential sensors for their functional expansion, connected architecture of smart masks for individual and group health care, and further considerations for actual deployment of such technology in the field. The details are as follows:

- Current status of existing commercial and academic smart masks
- Smart mask expansion in terms of personal health care and disease diagnosis
- Connected architecture and applications of smart masks
- Further real-world considerations

## Features and Applications of Current Smart Masks in the Field

Relevant smart masks available in the market were found through web searches, including Amazon, using the following search terms: “Smart Mask,” “Facial,” and “Electronics.” The search for publications was performed using 5 databases (Google Scholar, Web of Science, ScienceDirect, PubMed, and EBSCO) on the basis of the following combinations of search terms: “Smart mask” OR “Smart face mask,” “sensor,” “IoT,” AND “Healthcare.”

We defined 3 major inclusion criteria of reports on smart masks in this review.. Specifically, these criteria involve the following: (1) sensing: sensors attached to the mask; (2) actuation: functional manipulation of the mask; and (3) connectivity: communicating sensor data using mobile, cloud storage, or IoT-based networks. Only articles published between January 2020 and May 2022 were included to examine smart masks developed after the COVID-19 outbreak. Finally, the study selection procedure resulted in 12 smart mask products and 13 smart mask research prototypes reported in this study. Tables 1 and 2 list their functions and features, respectively. Detailed selection criteria are provided in Multimedia Appendix 1.

**Table 1:** Commercially available smart masks with their key features.

Name and purpose			Function		Feature
**Air control with respiration rate–sensing**					
	AO AIR Atmos mask [10]	Automatic fan control with respiration rate–sensing and filter status check		S^a^: Filter status and respiration A^b^: Fan on/off control C^c^: Bluetooth	
	LG PuriCare (2nd Gen) [11]	Automatic fan control with respiration rate–sensing		S: Respiration rate A: Fan on/off control C: Bluetooth	
**Ventilation**					
	ATMOBLUE Face Mask [12]	Three fan speed modes and air quality check		S: Air quality A: Fan speed control C: Bluetooth	
	Belovedone Air Purifier [13]	Two fan speed modes		A: Control fan speed	
	Philips Fresh Air Mask [14]	Three fan speed modes		A: Control fan speed	
	Xiaomi Purely [15]	Three fan speed modes		A: Control fan speed	
	CSE&L AIRVISOR [16]	Three fan speed modes		A: Control fan speed	
	CELLRETURN CX9 [17]	Sterilization and LED skin care		A: LED^d^ sterilization and skin care	
	Razer Zephyr [18]	Two fan speed modes and lighting		A: Control fan speed and customizable lighting zones C: Bluetooth	
**Communication aid**					
	CLIU Pro [19]	Air quality check and built-in microphone		S: Air quality, mask wear time, and head motion C: Bluetooth	
	Donut Robotics C-FACE [20]	Speech to text and voice translation		A: speech-to-text message, voice call, and translation C: Bluetooth	
	TrendyNow365 LED Mask [21]	Text display on mask surface		A: Display custom LED letters C: Bluetooth	

^a^S: sensing.

^b^A: actuation.

^c^C: connectivity.

^d^LED: light-emitting diode.

**Table 2:** Smart mask research prototypes from academic journals.

Name and purpose		Function	Feature
**External pathogen detection and elimination**			
	ADAPT [22]	Pathogen sensing and mist spray activation	S^a^: Airborne particle sensing A^b^: Mitigation module on/off C^c^: Bluetooth
**COVID-19 detection**			
	SARS-CoV-2-sensing face mask [23]	Detects COVID-19 infection	S: Paper-based nucleic acid diagnostics
	Lightweight and zero-power smart face mask [24]	Monitor cough and check mask-wearing	S: Mask deformation C: RF^d^ transponder
	AG47-SmartMask [25]	Monitor cardio-respiratory variables and to detect cough	S: Breathe pattern, skin/DSV^e^ temperature, humidity, air pressure, HR^f^, and SpO2^g^ C: Bluetooth
**Respiratory disease–monitoring**			
	Smart face mask with Heat flux sensor [26]	Noninvasive body temperature and breathing rate–monitoring	S: Facial skin temperature and breathing rate C: LoRa^h^ and Wi-Fi
	Smart facemask for wireless CO_2_ monitoring [27]	Monitor CO_2_ in DSV	S: CO2 concentration C: NFC^i^
	Smart face mask with ultrathin pressure sensor [28]	Breath monitoring	S: DSV pressure change C: Wi-Fi connection
	Smart face mask with wearable pressure sensor [29]	Breath monitoring	S: DSV pressure change C: Bluetooth connection
	Smart medical mask for health care personnel [30]	Detect respiratory breathing, fever, and alert possible face irritation	S: DSV temperature, mask strain C: Wi-Fi
	Lab-on-Mask [31]	Monitor cardio-respiratory variables	S: HR, BP^j^, SpO2, and skin temperature C: Bluetooth connection
**General health monitoring**			
	FaceMask [32]	Monitor cardio-respiratory variables and mask-wearing	S: Humidity, DSV or external temperature, volatile organic compounds. And head motion C: Bluetooth connection
	Facebit [33]	Monitor HR, respiration rate, mask fit, and wear time	S: HR, respiration rate, mask fit, and wear time C: Bluetooth
	Masquare [34]	Monitor cardio-respiratory variables	S: Respiratory pressure, HR, SpO2, and head motion C: Bluetooth

^a^S: sensing.

^b^A: actuation.

^c^C: connectivity.

^d^RF: radiofrequency.

^e^DSV: dead space volume.

^f^HR: heart rate.

^g^SpO_2_: blood oxygen saturation.

^h^LoRa: long range.

^i^NFC: near-field connection.

^j^BP: blood pressure.

Most commercial masks used in daily life provide actuations based on use, such as exchangeable filters, self-sterilizers, embodied microphones, and integrated fans. In total, 4 smart masks had sensing capabilities such as air pathogen check, filter status, and breath monitoring. In total, 11 smart masks included actuation with mostly inner fan speed control and LED lighting control. A total of 7 smart masks supported a connectivity feature through a Bluetooth connection with the smartphone. The masks that supported all 3 features (ie, sensing, actuation, and connectivity) were those of Atmos AO AIR [10], LG PuriCare (2nd Gen) [11], and ATMOBLUE [12]. These smart masks offer inner fan control actuation and Bluetooth connectivity while using different sensing (filter, respiration rate, and air-quality checks). Commercial masks have focused on mitigating discomfort such as breathing difficulty, excessive moisture inside the mask, fogging of glasses, and hygiene problems caused by long-term use [35-37]. Besides protection, the masks of CLIU [19], Donut Robotics [20], and TrendyNow365 [21] aimed to overcome speech problems with mask-wearing. Additional investigations, such as mask material, weight, and battery usage time, are presented in Multimedia Appendix 1.

While commercial smart masks were focused on user comfort, academic prototypes were designed for sensing capabilities such as health monitoring and disease detection. For example, in terms of COVID-19 detection, Nguyen et al [23] integrated a cell-free sensor to detect SARS-CoV-2, and Ye et al [24] and Fois et al [25] focused on detecting abnormalities such as coughing behavior. Not specific to COVID-19 but to cope with general respiratory disease, Lazaro et al [26], Escobedo et al [27], Zhong et al [28], Yang et al [29], Kim et al [30], and Pan et al [31] monitored breathing patterns. From a general health monitoring perspective, Gravina et al [32], Curtiss et al [33], and Fischer et al [34] monitored biosignals such as heart rate, respiration rate, and body temperature. Acquired sensor readings were then analyzed through smartphone apps for display.

All prototype masks were considered with regard to their physiological sensing capabilities. A total of 12 smart masks were considered with connectivity features using Bluetooth connectivity, near-field communication (NFC), a long range, and Wi-Fi connectivity with the smartphone. Ye et al [24] further demonstrated a radiofrequency (RF) feature using silver nanowires attached to the inner layer for monitoring cough and mask usage. Overall, the current features of smart masks available in the market offer environmental (air quality) monitoring, mask quality–monitoring, and functions for user comfort. On the other hand, research prototypes can be summarized as health monitoring and respiratory disease detection.

## Possible Directions for Feature Extension

Our investigation of research prototypes showed that existing masks support health monitoring and disease diagnosis on the basis of vital signs such as respiration, blood oxygen saturation, and body temperature. In this section, we further explore what other biosignals can be measured and what applications can be used through a smart mask as a wearable device for health care and safety. In addition, we argue that it is critical to reduce the posterior auricular (back of the ear) discomfort and pain caused by long-term wearing of the mask, as witnessed by a mask frame extension that supports an ear strap introduced recently [38]. In consideration of the ear strap frame, we would like to present a viewpoint on the extension of the application of the smart mask and its potential as a biosignal measuring device. To systematically search for feasible sensors, the expressions “smart” and “intelligent” textiles or “wearable electronic” are keywords used for selection. Sensors that sense and react to biosignals, environmental conditions, or stimuli, such as those from breath, skin, head motion, air, or other sources, were investigated. Multiple biosignal sources can be recorded around the face with sensors incorporated into the smart masks to measure biosignals and interior or exterior environmental factors [22,39].

For the facial part of the mask, pressure sensors can be used to obtain the respiration rate and inhalation volume to monitor breathing patterns [28,29]. These are piezoelectric-like sensors that are sensitive enough to respond to exhale volume pressure and flexible, lightweight, and energy-efficient circuits that can fit into the mask. With continuous monitoring of breathing patterns, we expect to observe users’ lung health or screen patients with chronic lung disease [40]. In addition to analyzing breath, chemical sensors can be used as markers for personal health problems and respiratory diseases by targeting specific molecules [41-48]. These sensors are based on metal oxides whose target compounds can be easily switched with specific reagents. Several applications include acetone for diabetes [41-43], hydrogen sulfide for small intestinal bacterial overgrowth [44,45], and toluene for lung cancer diagnosis [46-48].

As the mask directly contacts the facial skin, a photoplethysmographic (PPG) sensor can be adopted to conduct pulse oximetry and measure heart rate variability, oxygen saturation, and blood pressure. These metrics are widely researched for indirect measures of physical and mental health [49-51], physical stress [31,49], and hypertension or hypotension [51-53], respectively. Electrooculography (EOG) [54,55], electrodermal activity (EDA) [56,57], and electromyography (EMG) [50,52,53] can also be adopted to measure various biophysical signals that arise from facial skin. For example, eye-blinking EOG measures have been linked to attention [58] and may infer the user’s mental state. The electrodermal response from EDA and facial muscle activation from EMG can be used as a measure of emotion such as anxiety or depression [55-57,59]. In addition, facial surface EMG was adopted for monitoring pain through facial expressions [60] (Table 3).

**Table 3:** Possible sensor integration on the masks.

Sensors and features						Applications
**Location: mask main body**						
	**Type: biosignal information**					
		**Source: breath (respiration)**				
			**Pressure sensor**			
				Respiration rate or volume	Personal health or sport [27,28]	
			**Chemical sensor**			
				Ketone: acetone	Personal health or disease (diabetes) [41-43]	
				Hydrogen sulfide	Personal health [44,45]	
				Toluene	Personal health or disease (lung cancer) [46-48]	
		**Source: facial blood vessels**				
			**Photoplethysmography sensor**			
				Heart rate variability	Physical health or mental health [49-51]	
				Oxygen saturation	Physical stress [31,49]	
				Blood pressure	Hypertension or hypotension [51-53]	
		**Source: skin**				
			**Electrooculography sensor**			
				Eye blink	Concentration [54,55]	
			**Electrodermal activity sensor**			
				Electrodermal response	Emotion [56,57]	
			**Temperature sensor**			
				Temperature change	Communicable diseases [25,26,31,32]	
			**Electromyography sensor**			
				Facial muscle	Emotion [55,59]	
				Facial muscle	Pain [60]	
		**Source: head**				
			**Inertial measurement unit**			
				Motion	Posture [61]	
	**Type: environmental information**					
		**Source: air**				
			**Chemical sensor**			
				Environment air quality	Local air quality [22,39]	
		**Source: external temperature**				
			**Thermometer**			
				Temperature	Local temperature [62]	
		**Source: external humidity**				
			**Humidity sensor**			
				Humidity	Local humidity [63-65]	
**Location: mask support frame**						
	**Type: biosignal information**					
		**Source: ear**				
			**Electroencephalography sensor**			
				Brain activity	Drowsiness or fatigue [66]	
		**Source: neck**				
			**Inertial measurement unit sensor**			
				Motion	Posture [61]	
			**Electrocardiographic sensor**			
				Heart	Heart disease [67,68]	

**Figure 1:**
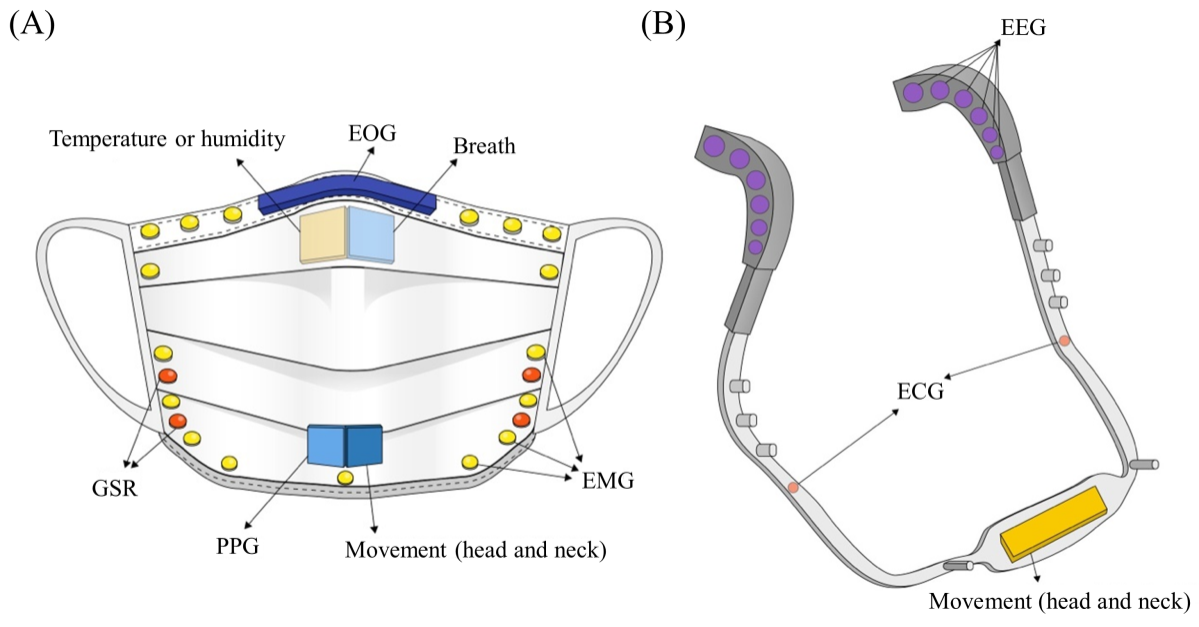
The possible sensor attachments on (A) a facial mask and (B) the ear strap. ECG: electrocardiography; EEG: electroencephalography; EMG: electromyography; EOG: electrooculography; GSR: Galvanic skin response; PPG: photoplethysmography.

A smart mask can also measure air pollution and several other environmental variables such as air quality [22,39], temperature [62], and humidity [63-65]. The inclusion of sensing air-tightness and the quality of filters can help ensure the additional benefits of smart masks by improving safety by providing an air-tight fit around the face. If the mask uses a support frame, such as a head or neck strap, electroencephalography (EEG) and electrocardiography (ECG) sensors can be applied to measure the electrical activity of the brain and heart. EEG signals have been used to detect a user's fatigue or drowsiness like fatigue in driving [66]. Integrating ECG can be an advantage over PPG readings as it records the heart’s electrical activity at its source [67,68]. Lastly, inertial measurement unit sensors can be attached to the ear strap for activity sensing that can discern fall or head collision [61]. The possible sensor attachments on a facial mask and ear strap are depicted in Figure 1.

## Toward Connected Smart Masks

**Figure 2:**
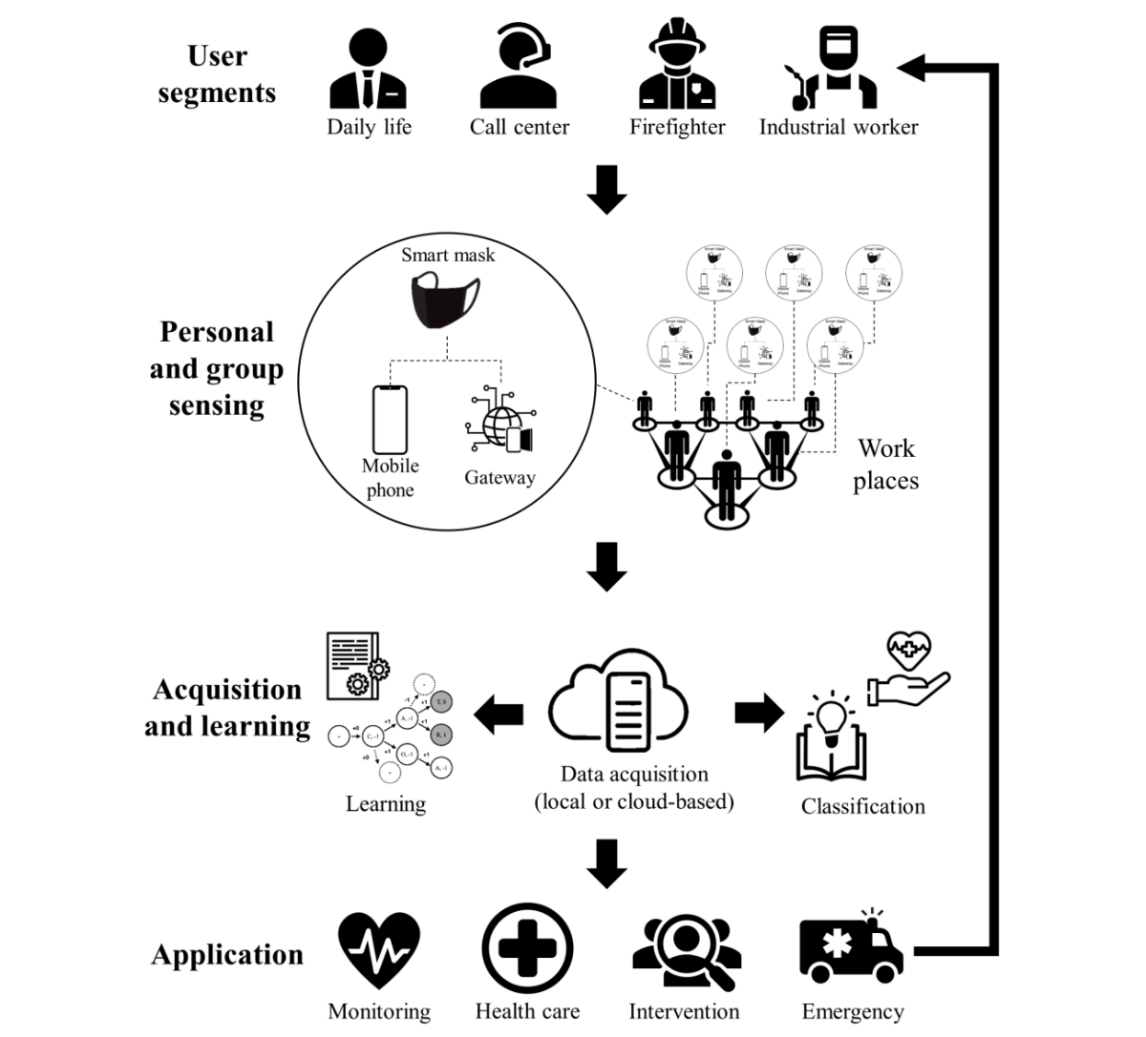
Connected smart mask architecture.

In this section, we attempt to seek opportunities beyond personal protective equipment to group management, so-called group-sensing, through connected smart masks as wearable devices for health care and safety. The advantages of group-sensing include continuously measuring and managing a population's physical and mental health through the sensors inside the smart mask or via connected smart mask platforms. Such advantages are particularly useful in dealing with infectious diseases that spread through contact and saliva, such as COVID-19 [69]. The smart masks of those at risk can be managed, and remote caregiving can be supported via connected devices. As in a prior study on smartwatches [70], their everyday health conditions (eg, breathing and heart rates) can be tracked and analyzed to detect early signs of respiratory behavior changes, which could be related to COVID-19 infection. Namely, beyond a personal sensing application, a group or community sensing application may share an aggregate version of information with the broader population. The architecture of connected masks is shown in Figure 2 by extending prior mobile sensing architecture [71,72].

For group sensing, the smart mask should be able to transmit the collected data to the server by using wireless communication protocols such as Wi-Fi, long-term evolution, 4G and 5G networks, Zigbee, and narrowband IoT without manual operation [73]. Besides, the analysis results should allow the user to take action or receive an alarm related to a particular hazard. Most smart masks integrate communication modules to use smartphones for displaying sensing results and as a gateway terminal to interact on the web [27,33,74,75]. In addition, smartphones allow short-distance connections such as Bluetooth, NFC, and radiofrequency identification, where acquired data can be transferred to local IoT gateways [73]. Furthermore, server clouds and relevant analytics technology are required to store smart mask data and process large sets of data to develop applications such as health care, safety monitoring, and intervention for the users. This kind of collaborative sensing will also address the challenges of individual sensing, such as reliability and coverage [76].

The information gathered in cloud servers can be used with machine learning (ML) and data mining applications [75,77]. The advantages of utilizing ML for group sensing results are system optimization and acquired data processing [77]. For instance, collecting data on device failures, usage time, filter, and battery can be analyzed for design considerations and maintaining the optimal operation of a smart mask. Furthermore, Gravina et al [32] reported the application of ML in smart masks, where they tested mask wear classification from sensor signals. In terms of data mining, a more detailed air quality map can be created as the user wears a smart mask with environmental sensors and moves around places collecting data. Moreover, GPS for community sensing can facilitate real-time sensing and location-based monitoring of masks and actions of multiple users in some local environments, such as COVID-19 contact-tracing, local airborne pathogen detection, or emergency services.

With modern technological advances, it has become possible to collect big data and create new knowledge that we have not been able to analyze before. Unlike conventional wearable devices, smart masks can collect biomarkers of respiration or the respiratory system and expand further knowledge on wearables. Previous work by Curtiss et al [33] and Hyysalo et al [75] shows detailed aspects of the connected smart mask platform and deployment considerations. Curtiss et al’s [33] Facebit smart mask accompanies a mobile app that displays sensing results such as heart rate, respiration rate, mask fit, and wear time. This app communicates with Facebit through Bluetooth and stores data in a local database. For now, stored data are used to track a user’s mask-wearing time and send a notification to replace the mask. As an open-source smart mask research platform, this work demonstrates proof-of-concept connected smart masks and presents further research on personalizing algorithms and applications for respiratory health tools. Hyysalo [75] illustrated the software architecture of the smart mask platform, including the mask, mobile app, and backend health artificial intelligence. In addition, this study envisioned a smart mask ecosystem [78,79]—a collection of infrastructure, analytics, and applications, to draw personal health trajectories.

## Further Considerations for Real-World Use

Lastly, we present and discuss viewpoints on the application fields of the connected mask and further considerations for practical use. As the smart face mask is a promising respiratory monitoring tool, we explored relevant fields where it can benefit direct needs. Aside from the primary field of daily-life health-monitoring, we envision several real-world uses such as sports training, ambulatory setting, industry and firefighter safety masks, and military applications. In the following sections, several directions for real-world deployment scenarios of smart masks are first discussed. Thereafter, we discuss sensor accuracy and reproducibility issues, most critical ones in measuring biosignals through all wearable devices. Ergonomic design for the general population needs to be considered for public acceptance of smart masks. Finally, privacy-aware data-handling is necessary for security to collect and manage personal biosignals.

## Service Application for Real-World Use

### Daily Life Health Monitoring

The smart mask presents an opportunity to apply advanced analytics to health care. The analysis of physiological changes, such as breathing pattern, pulse rate, and tidal volume, enables us to monitor respiratory health, diagnose relevant diseases, and point of care through continuous monitoring. In addition, other various features can be obtained, as we discussed in the possible sensor extension scenarios, for instance, stress and fatigue [80].

### Sports Training

In particular, smart masks can be adopted for measuring the cardiopulmonary exercise load, which is an important index in evaluating exercise capacity. Previously, this was done by wearing additional equipment in wired or wireless form with controlled settings [81]. This test can be easily accessible to the general population; for example, in a gymnasium or through home-based training through smart mask application. Furthermore, owing to the recent COVID-19 pandemic, there is increasing demand for indoor exercise platforms such as Zwift [82], where individuals can virtually compete with users on the internet and measure exercise ability and improvements. The smart mask can contribute as a wearable device for additional exercise measures in such settings.

### Emergency Room or Ambulatory Settings

In the emergency room or ambulatory settings, masks have been used to deliver air and monitor respiration. We expect smart masks to be adopted to track health status without any additional device. Additionally, nosocomial infections, such as ventilator-associated pneumonia, can be detected with the use of the smart mask [83].

### Industry Workers and Firefighters

Many workers at coal mines, construction sites, and chemical plants and firefighters at fire scenes are prone to hazardous gas; thus, wearing a mask is mandatory for safety issues. Smart masks can be used to track the health status of people who have been poisoned by gas or toxic substances or have been exposed by measuring the surrounding situation. Besides, real-time environmental monitoring can ensure user safety and prompt responses to fast-changing hazardous events through the detection of gas leakage or toxic events [84].

### Soldier Safety and Survivability

Recently, there has been ongoing research on wearable devices such as vests and helmets to collect biosignals for the safety and survival of soldiers [85]. The smart mask can also be a promising wearable device in respiratory monitoring. It is expected that safety and survival can be further improved by collecting the soldier’s biosignals, location information, or information about the surrounding environment. These measures help monitor the soldier's physical and mental health status and decision-making. Moreover, breath analysis can predict and monitor the onset of pulmonary injury due to various environmental and infectious exposures [86].

## Accurate and Reliable Sensors

One major requirement for such predictive diagnostics is that sensor information must be accurate and reliable. The type of sensor and its placement affect the measurements. For instance, potential inaccuracies rise with excessive motion artifacts involving many physical activities, such as sports, firefighting, or military action. Although the reviewed articles described potential applications and demands for health intervention, they provided little evidence related to the usability and practicality of the proposed device. As the temperature and humidity rise owing to mask-wearing, the adhesion between the sensor and the skin may decrease, and sweat generated by humidity may negatively affect accurate sensor signal measurement. Beyond sensing accuracy and reliability, it is important to consider additional metrics, such as smart mask interoperability, versatility, power consumption, and durability, to examine the usefulness of the system as well as comfort and ease of use for different population characteristics [87,88].

## Ergonomic Design for Usability

If users wear heavy equipment such as a helmet for a long time, it can strain their head and neck [89-91]. Masks with smart functions also increase in weight, unlike existing masks, owing to the addition of batteries, sensors, and fans. Therefore, it places a burden on the head and neck and may cause deformation in posture. If the systems within smart masks became more complicated, these could become more uncomfortable and make users reluctant to wear them. Detailed surveys on usability and performance evaluation from daily life trials need to be conducted to ascertain the usability of smart masks [92-94]. Maximizing and optimizing the battery lifetime of the smart mask ensures user satisfaction and comfort [95,96]. If the device supports recharging, the rechargeable battery of the mask is a major contributor to the mask’s weight. If the communication between the smart mask and the smartphone requires much energy and acquiring data from sensors may rapidly drain the battery, a larger battery capacity is then required. Thus, the overall weight of the mask increases. Therefore, in developing a smart mask, it is necessary to consider the battery size and material related to weight. In addition, since the material of their mask is in contact with the skin surface, it is necessary to use an approved suitable material [97]. Overall, the potential reluctance of users can be reduced by incorporating simple protocols for the number of sensors and user specificity, comfort, including weight, and fashion considerations for the general population [98,99].

## Privacy-Aware Data-Handling

One challenge in developing connected smart mask architecture systems is the collection of personal information and privacy infringement. With the advancement of the IoT, real-time monitoring data are shared and analyzed to identify factors related to events. Although this monitoring is intended to assist users, some aspects of personal privacy are violated [100-104]. Prior studies have shown that privacy concerns related to wearable cameras are often influenced by users’ social, behavioral, and environmental contexts [105]. For example, wearable camera users are often conscious of bystander privacy, and likewise, bystanders are concerned about potential privacy violations (eg, subtleness and ease of recording) [106]. In addition, advanced data processing methods may have privacy implications. For instance, personal physiological data or location information can be misused because of poor data management policies. In these scenarios, health monitoring results may encourage the tracking of work performance (ie, using the data for secondary purposes without explicit consent). This practice may influence the review of workers’ performances and may cause monitoring to become a surveillance practice beyond health monitoring. Beyond secondary use, the security of the devices themselves can also be problematic, as the low computing power within smart mask systems may make them vulnerable to unauthenticated access [107,108]. As smart mask technology is still in its infancy, these implications are not yet fully understood and should be considered in future implementation strategies.

## Conclusions

This study examined recent smart masks in conjunction with accompanying systems that could be used to prevent COVID-19 and other respiratory diseases. We then offered our viewpoints on smart masks in the form of emerging IoT solutions. Reviewing commercially available smart masks revealed the trend that smart masks were mainly designed to address user discomfort. However, recent research prototypes were taking further steps, not only dealing with COVID-19 but toward general health monitoring by supporting breathing and physiological signal sensing. Thus, we sought further functional expansion on smart masks by investigating previous mobile sensing studies. In addition, we extensively discussed novel opportunities for group health management through a connected smart masks platform. We believe that smart masks can serve as a truly cutting-edge device that expands the coverage of health monitoring and helps reach the next level of wearables.

## Appendix

Multimedia Appendix 1 Table showing specifications of 12 commercially available smart masks.

## Abbreviations

EDA: electrodermal activity
EMG: electromyography
EOG: electrooculography
IoT: Internet of Things
KAIST: Korea Advanced Institute of Science & Technology
ML: machine Learning
NFC: near-field communication
PPG: photoplethysmography
