# Supplementary material for: Beyond Pathogen Filtration: Possibility of Smart Masks as Wearable Devices for Personal and Group Health and Safety Management
Source: JMIR Mhealth Uhealth. 2022 Jun 21;10(6):e38614. doi: 10.2196/38614 (PMC9217147; doi:10.2196/38614)
Supplement: Multimedia Appendix 1 [file mhealth_v10i6e38614_app1.docx]

**Appendix Tables**

**Appendix Table S-1.** Commercially available smart masks (S: sensing; A: actuation; C: connectivity; LED: Light-emitting diode; HEPA: High-efficiency particulate absorbing filter; N/A: not applicable).

| **Name** | **Mask material** | **Filter** | **Function** | **Feature** | **String type** | **Note** | **Weight** | **Price ($)** | **Battery** |
| --- | --- | --- | --- | --- | --- | --- | --- | --- | --- |
| Atmos  AO AIR [10] | Hypoallergenic materials including silicones | Composite construction  (1 month) | Automatic fan control with respiration rate, filter status check | S: Check filter status and respiration rate  A: Fan On/Off control  C: Bluetooth | Head-mounted device | -- | 290 g | 350 | 5 hr |
| LG  PuriCare (2^nd^ Gen) [11] | Medical-grade silicone | H13 grade HEPA filter  (1 month) | Automatic fan control with respiration rate sensing | S: Respiration rate  A: Fan On/Off control  C: Bluetooth | Adjustable ear loops | -- | 145 g | 249 | 8 hr |
| ATMOBLUE  Face Mask [12] | Silicone interface | HEPA H13 filter (last up to 6 weeks) | Three fan speed modes,  air quality check | S: Air pollution check  A: Fan speed control, airflow control  C: Bluetooth | Omni-directional head strap | -- | 190 g | 199 | 8 hr |
| Belovedone Air Purifier [13] | Food-grade silicone | Four-layer filter | Two speed modes | A: Control fan speed (two steps) | Thicker loops on head | -- | 80 g | 29.99 | 4–8 hr |
| Philips  Fresh Air Mask [14] | Breathable meshes(body), aluminum (air module) | Carbon filter  (up to 122h) | Water-resistant,  three wind modes | A: Control fan speed (three steps) | Normal string to ears | Washable | 300 g | 199 | 2–3.5 hr |
| Xiaomi  Purely [15] | Nonwoven fabric, nanometer electret fiber | Nanofiber electret filter | Three speed modes | A: Control fan speed (three steps) | Normal string to ears | Detachable design | 50.5 g | 32.99 | 4–8 hr |
| CSE&L  AIRVISOR [16] | Silicon body | Copper filter  (7 days) | Three speed modes | A: Control fan speed (three steps) | Head strap | -- | 125 g | 70 | 8 hr |
| CELLRETURN  CX9 [17] | Silicon | (1–7 days) | Sterilization,  LED skin care | A: LED sterilization,  skin care | Adjustable ear strings and headband strap | Washable | 130 g | 299 | 1.5 hr |
| Razer  Zephyr [18] | Medical Grade BPA-Free Silicone | Meltblown filters  (3 days) | Two fan speed modes, lighting | A: Control fan speed, customizable lighting zones  C: Bluetooth | Adjustable ear loops | -- | 206.1 g | 99.99 | 3.5-8 hr |
| CLIU Pro [19] | Silicone,  magnetic frame | Interchangeable carbon filter | Microphone,  Bluetooth,  accelerometer | S: Aerosol sensor for virus, breathe, air  C: Bluetooth | Adjustable silicone band on head | Trans-parent mask | 200 g | 298 | N/A |
| Donut Robotics  C-FACE [20] | Soft plastic and silicon cover | -- | Speech to text, voice translation | A: speech to text message,  voice call, translation  C: Bluetooth | -- | Fit to other strap masks | N/A | 40 | 1 hr |
| TrendyNow365 LED Mask [21] | Cotton | Carbon-activated filter | Text display | A: Display LED letters  C: Bluetooth | Adjustable ear loops | -- | 65 g | 19.99 | 8 hr |

The specifications were based on the data available on the vendor's webpages.
